# Reframing healthy food choices: a content analysis of Australian healthy eating blogs

**DOI:** 10.1186/s12889-019-8064-7

**Published:** 2019-12-19

**Authors:** Rebecca Mete, Jayne Curlewis, Alison Shield, Kristen Murray, Rachel Bacon, Jane Kellett

**Affiliations:** Faculty of Health, The University of Canberra, University Dr, Bruce, ACT 2617 Australia

**Keywords:** Healthy eating blogs, Communication, Dietitian, Social media

## Abstract

**Background:**

Blogs are widely being used by health professionals and consumers to communicate and access nutrition information. There are numerous benefits for dietitians to establish and contribute to healthy eating blogs. In particular, to disseminate evidence-based nutrition information to promote healthier dietary practices. The aim of this study was to explore characteristics of popular healthy eating blogs and inform the provision of healthy eating information in the Australian context.

**Methods:**

A content analysis approach was used to identify characteristics of popular Australian healthy eating blogs. A purposive and snowball sampling approach was used to identify healthy eating blogs from search engines including Google, Bing and Yahoo. Blogs were deemed eligible if: (1) the author self-identified as a health professional; (2) the blog was written by a single author; (3) the blog was written by an Australian author; (4) the blog had a minimum of one post per month, and (5) the blog focused on communicating healthy eating information to the general adult population.

**Results:**

Five popular blogs were followed over a three-month period (December 2017–March 2018), with 76 blog posts included for analysis. Characteristics of these popular blogs were examined and four main features were identified: (i) clearly conveying the purpose of each post; (ii) developing a strong understanding of the reader base and their preferences; (iii) employing a consistent writing style; use of vocabulary and layout; and (iv) communicating healthy eating information in a practical manner. These findings reveal important insight into the features that promote effective nutrition communication within this context.

**Conclusion:**

Findings from this study highlight common characteristics of popular healthy eating blogs. Future research into the development of blog guidelines which incorporate the characteristics identified in this study can support dietitians in establishing or contributing to the successful provision of evidence-based nutritional information through blogs.

## Background

In Australia, it is estimated that 83% of the population have access to and use the internet [1, 2]. In 2011, the Pew Research Centre reported that seven in ten adults searched for health-related information online [3]. The same report also indicated that from a sample of 2065 internet users, 44% specifically searched for nutrition-related information [3]. Additionally, it has been suggested that consumers are proactively seeking online nutrition-related information with social networking sites being a preferred platform to access this information [4–6]. Therefore, to reach and communicate health and nutrition information to consumers online, health professionals are commonly utilising social networking sites, including Facebook, Instagram, Twitter, and blogs [7].

Blogs are increasingly growing in interest and popularity, and are commonly defined as websites containing posts which are presented in reverse chronological order [8–10]. Health-related blogs are currently used in a variety of ways including as a tool for health promotion, for discipline-focused information provision, to offer support for individuals with chronic disease, as a carer support network, a tool to understand mental health concerns, and to explore motivations and expectations of individuals [11]. A recent review of social media practices within the dietetic profession identified discussion forums, blogs and other social networking sites to be common tools for the dissemination of nutrition information [12]. Whilst blogs are being increasingly used by dietetic and health practitioners, they are also becoming an accepted platform for accessing nutrition information by consumers [9]. This is important as blogs focused on communicating knowledge about healthy eating have the capacity to reach a diverse sample of consumers who regularly use the internet [7–10]. In addition, there are many benefits for dietitians to establish or contribute to blogs including to promote healthier food choices and dietary behaviours [11–14], as an inexpensive means of communication [15, 16], and for the continual access to evidenced-based nutritional information for the consumer [17].

Disseminating healthy eating information in an effective manner is integral in informing consumers about healthier dietary choices to reduce chronic disease risk [14]. Previous research has focused on factors that influence the effectiveness of communicating healthy eating educational materials on websites and in print media, rather than in healthy eating blogs specifically [18–21]. Factors influencing the effectiveness of educational material include readability, writing style [18–20, 22–24] and appropriate use of vocabulary [21]. To account for poor literacy levels, there is consensus within the literature that education materials should be written to the standard of a sixth-grade reading level (equivalent to an 11–12 year old child with six years of education) [21, 22, 25]. Writing style is also an important factor, and it is recommended that an active and conversational tone should be adopted [24]. This recommendation is believed to encourage the reader’s interest through the use of familiar language and also increases readability [24]. In addition, it has been suggested that appropriate use of vocabulary is an essential feature when communicating written education, as it is recommended that educational materials should not use technical jargon and abbreviations, as unfamiliar terms can deter the reader [24]. This study explores characteristics of popular healthy eating blogs and the ways in which healthy eating messages are communicated within the Australian context. This study also aims to offer suggestions for how this information can be communicated to guide dietitians in creating popular (successful) healthy eating blogs.

## Methods

### Overview

This study was conducted using a content analysis approach to identify characteristics of popular healthy eating blogs. Content analysis is a research method that has been increasingly used to analyse digital content, including written and visual content in internet forums, websites and in social media platforms [26–29]. Australian healthy eating blogs authored by self-identified health professionals were identified and followed for three months (December 2017–March 2018).

### Sampling and selection

**Fig. 1:**
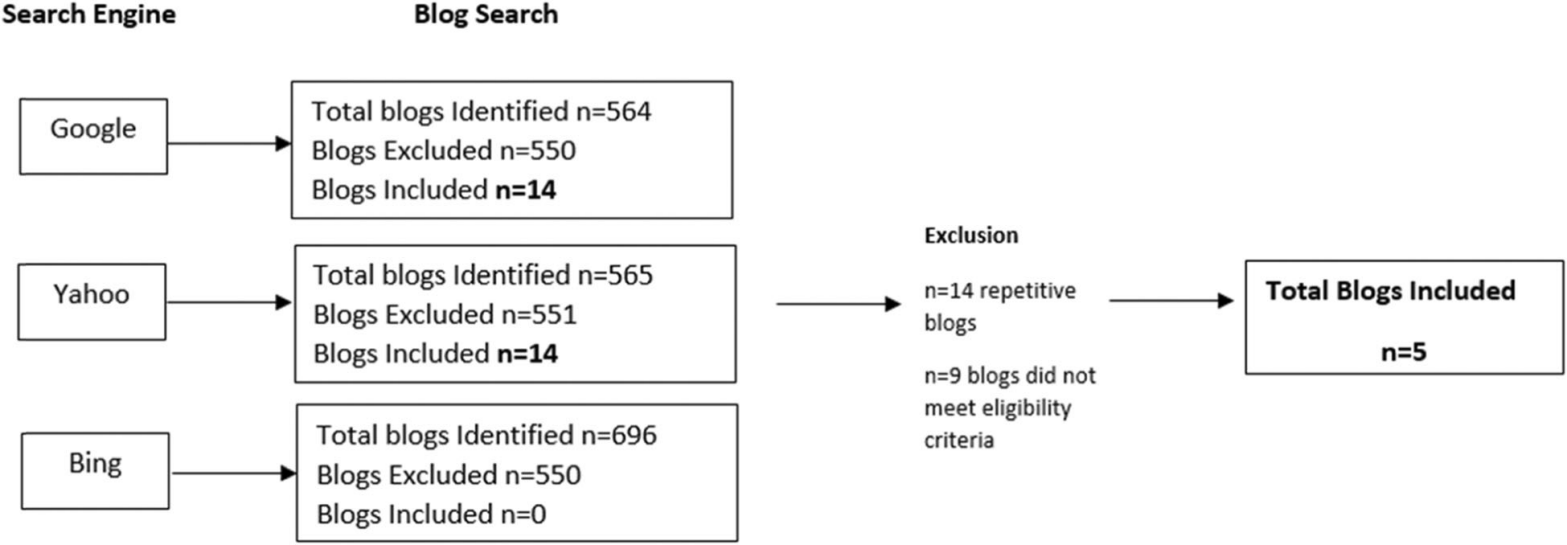
Blog Identification Strategy

A purposive and snowball sampling approach was used to identify healthy eating blogs from search engines including Google, Bing and Yahoo (Fig. 1). The search terms used in each search engine included ‘Australian Healthy Eating Blogs’ and ‘Top 100 Australian Healthy Eating Blogs’. There is no precise method to estimate the number of online healthy eating blogs [30, 31]. Therefore, blogs were identified by focusing on the first page of each search engine, as it was assumed they are the most widely read and influential [30–32]. This decision was driven by a study conducted by Jansen and Spink (2009) that investigated consumer search engine click-through behaviour and reported 73% of consumers did not move beyond the first page of search engine results [32]. Additionally, current knowledge of search engine optimization (SEO) suggests that first page search engine results have higher website traffic [33].

Each webpage included was systematically searched and assessed for eligibility. Healthy Eating blogs were deemed eligible if: [1] the author self-identified as a health professional [2]; the blog was written by a single author [3]; the blog was written by an Australian author [4]; the blog had a minimum of one post per month, and [5] the blog focused on communicating healthy eating information to the general adult population. Blogs were excluded if they focused on a specific condition or disease state, if they could not be accessed due to a broken web link or if they were privately accessible. Additionally, micro-blogs which are defined as short posts restricted to 140 characters, were not included for analysis, as the primary focus of this study is to explore blog characteristics and the ways in which healthy eating messages are communicated [34, 35]. Due to the character restrictions, the intended purpose of micro-blogs is to provide brief updates [34, 35].

In this study, authors who identified as a health professional were chosen because they are already an accepted source of nutrition and health information. It was assumed that blogs written for the exclusive purpose of providing healthy eating information by health professionals would aim to provide evidence-based information, which may not be case for personal blogs. Health professionals in this study included, Accredited Practicing Dietitians (APDs), Nutritionists, Wellness and/or Health Coaches, Personal Trainers, General Practitioners, or any combination of the above. The qualifications of authors were self-reported and recorded on the ‘about me’ section of each blog; they were not further verified by the research team.

### Procedure

A total of 14 healthy eating blogs were identified for analysis. Each blog was routinely viewed over the three-month collection period to ensure blogging frequency met inclusion criteria (9 blogs did not meet criteria and were subsequently excluded). Additionally, due to the temporary nature of the Internet and to ensure no adaptations to posts occurred, screenshots were taken of each blog post and stored as separate Word Documents.

### Ethics

The study was approved by the relevant institutional Human Research Ethics Committee [removed for blind peer-review]. Conducting internet research presents challenges that need to be acknowledged, including the boundary between public and private content in relation to consent [36]. Hookway (2008) suggests that ‘accessible blogs may be personal but they are not private’ [37]. In this study, blogs were considered public if they were not privately accessible or password protected, therefore the institutional ethics committee did not require individual consent from blog authors. However, to preserve anonymity, blogs were de-identified for the purpose of the content analysis and reporting.

### Data analysis

A coding scheme was derived from previous research on the content of information communicated within health websites [19, 20, 38, 39]. The Health-Related Website Evaluation Form (HRWEF) and the Suitability Assessment of Material (SAM) were adapted to create a coding scheme to help guide analysis [19, 20, 38–41]. The HRWEF evaluates the content, credibility, currency, accuracy, reliability, readability and design of health-related websites, based on a scoring system [19, 20, 38, 39] . The SAM tool evaluates literacy, readability, writing style, vocabulary, context, graphics and illustrations, layout, typography, interaction and cultural appropriateness of health-related websites [19, 20]. Each variable was coded through a scoring system which reflected the degree to which they aligned with criteria adapted from the HRWEF and SAM. A priori content analysis was conducted on four blog posts from identified healthy eating blogs that were dated prior to the commencement of data collection to ensure applicability of the coding scheme and for coding training purposes.

Coding was conducted by two researchers [blinded for peer-review] using Microsoft Excel. Both researchers had formal University training within the field of Nutrition and Dietetics. Coding was undertaken collaboratively to aid reliability and consistency of the coding process. Each blog was assigned an identification letter and each post within a blog was assigned with an identification number. This allowed for the two researchers to discuss and compare coding during analysis. Differences in code assignments were resolved by the two researchers by discussing the rationale behind the coding decision and determining an agreed coding assignment. Additionally, in order to facilitate transparency, researchers documented their own thoughts about the purpose of the blog post at the completion of analysis which were then collaboratively shared between the researchers.

## Results

### General blog characteristics

A total of five blogs were followed over a three-month period from which 90 blog posts were identified. From the 90 blog posts, 14 blog posts were excluded due to content being non-nutrition related, resulting in a total of 76 blog posts for analysis. Professions that were identified included, a Nutritionist, Naturopath, Personal Trainer or Wellness Coach, with all authors reporting a single profession except for one blog where the author indicated multiple professional titles. The frequency of posting by authors varied (10–21 posts), however on average authors posted 15 times over the course of three months.

Successful healthy eating blogs were defined in this study as those listed on the first page of a major search engine (Google, Bing and Yahoo) [30–32]. Healthy eating blogs identified by the search strategy did not include those authored by self-reported APDs. Despite healthy eating blogs being written by different authors, there were common characteristics between blog posts. These common characteristics included: (i) clearly conveying the purpose of each post, (ii) an understanding of the reader, (iii) consistent use of writing style, vocabulary and layout; and (iv) communicating healthy eating information in a practical manner (see Table 1).

**Table 1:** Quantitative content analysis of 76 blog posts

Assessment Criteria	% ^(a)^
Purpose	
Purpose explicitly stated	97%
Purpose implied	3%
Content Knowledge	
Procedural knowledge (90–100%)	64%
Mainly Declarative with at least 40% focusing on procedural knowledge	17%
Declarative Knowledge (90–100%)	11%
Not applicable	8%
Adherence to the Australian Dietary Guidelines	
Explicitly adheres	43%
Somewhat adheres	17%
Does not adhere	7%
Not applicable	33%
Writing Style	
Conversational and active voice	100%
Somewhat conversational and active	0%
Passive voice	0%
Vocabulary	
Use of common words, technical jargon explained and use of imaginary	100%
language	0%
Use of common words and technical jargon not explained	0%
Use of uncommon words and extensive jargon is used	
Layout	100%
Illustrations are used, layout is consistent, visual cues, adequate white space and appropriate use of colour	
Some criteria of the above are present	0%
Layout is uninviting or hard to read	0%

^**(a)**^ Percentage of blog posts relating to each criterion

Most blog posts focused on a single targeted healthy eating message, directly in line with the purpose of the post, rather than containing multiple healthy eating messages. Authors communicated the purpose early in the blog post using catchy headings and within the first few sentences of the first paragraph. Blog post headings were in the style of practical ways to increase fruit and vegetable intake; simple time saving food preparation recommendations; and practical ways to improve or add excitement into mealtimes. Readers were given a clear sense of the purpose of the post and this established expectations.

Findings indicated that authors conveyed a sense of understanding within blog posts to their reader’s needs. This was identified by the author directly acknowledging reader comments and concerns as the basis for a particular post. Communication was also encouraged by the author through statements that encouraged and directed the reader to provide feedback using the comment box*.* However, despite author encouragement, there were differences between reader participation using the comment box between blogs (see Table 2). Reader participation using the comment box ranged from 14 to 100%, with only Blog E achieving reader participation in all blog posts posted.

**Table 2:** The number of blog posts containing comments

Blog ID	Total Blog Posts (over the 3 months)	Total # of Blog Posts that contained comments	% of Posts during the 3 months that were commented on
A	21	13	62%
B	11	2	18%
C	14	2	14%
D	20	2	10%
E	10	10	100%

The writing style, vocabulary and layout of posts also appeared to encourage a relationship between the author and the reader. Posts were written in a conversational manner, with technical jargon explained using metaphors and simple language. Authors commonly wrote in the first person and positioned themselves as similar to their readers as an individual who also faces positive and challenging food and nutrition-related experiences. Example statements include: *‘… I’m passionate about healthy eating …’ (Blog A)* and *‘I’ve personally watched [removed to de-identify]’ (Blog B).* Both the conversational writing style and use of simple vocabulary complemented the translation of nutrition knowledge by allowing readers to understand nutrition-related concepts in non-technical language. In addition, blog posts focused on conveying a positive message about the benefits the food(s), rather than framing topics in negative terms.

Blogs incorporated the use of a consistent visual layout and structure from post to post within each blog. Commonly the visual layout of a post guided the reader’s attention from a bolded heading (emphasising the purpose of the post) to a complementary eye-catching photo directly below. The reader was guided towards the introduction of the post which was usually accompanied by another visual cue separating the introduction from the body of the post. The body of the post commonly focused on communicating healthy eating information. The reader was then guided to the end of the post which was identified by the authors’ sign off and encouragement to communicate via the comment box.

It was found that 64% of blog posts communicated healthy eating information in a practical manner by using recipes, practical tips and author recommendations (see Table 3). Typically, these techniques would be integrated into the structure of a post. Posts would commonly begin with an introductory paragraph which would state the purpose and/or associated health benefit(s). This would lead onto the next paragraph which would incorporate personal narratives or opinions from the author’s perspective, with practical information then provided. Recipes were used to communicate healthy eating messages in several ways including: a solution for readers to incorporate a specific food with health benefits into their diet; providing healthier alternatives to a dessert or snack; and food alternatives catering for a variety of food preferences. Recipes were influenced by season and the latest food trends, for example: coconut oil, maple syrup, rice malt syrup, avocado, organic coconut sugar, spelt flour and apple cider vinegar were incorporated into recipes. Vegetarian recipes were commonly posted along with current trends and ‘superfoods’, including ‘bowls’ (e.g. Buddha bowls, smoothie bowls), ‘smoothies’ (e.g. green smoothies, matcha smoothies) and ‘guilt free’ desserts (paleo bars, raw bars); further highlighting the up-to-date nature of blog posts. Practical tips and recommendations were commonly communicated in a straight-forward manner by bullet points or sequential steps. Recommendations were not exclusive to nutrition and often incorporated behavioural and motivational recommendations. For example, ‘*Make healthy swaps*’ (nutrition-related)*, ‘Inspire your friends and family’* (motivational) *and ‘Don’t deprive yourself’* (behavioural) (Blog D).

**Table 3:** Nutrition communication methods used in blog posts

Frequency	Method
38	Recipe (including recipe only, recipe and suggested variations for allergies & recipe and health benefit information)
15	Practical tips (including: 5 top tips, steps to follow, food lists, bullet point summaries)
11	Author recommendations (including personal experiences and guest interviews)
8	Individual food focus (including healthier food swaps & descriptive information)
4	Meal ideas (including cooking tips & flavour combinations)

A comparison between healthy eating information from blog posts with recommendations from the Australian Dietary Guidelines (ADG) suggested that only 43% of information clearly aligned with recommendations (see Additional file 1: Table S1). Most of the healthy eating information aligned with guidelines two and three, *‘Enjoy a wide variety of nutritious foods from the five food groups every day’* and *‘Limit intake of foods containing saturated fat, added salt, added sugars and alcohol’,* respectively [42]. Posts often encouraged the consumption of fruit, vegetables, legumes and lean meats; and encouraged the concept of moderation, whilst permitting the occasional consumption of foods that do not fall within the five food groups.

## Discussion

This study identifies the main features of a popular healthy eating blog, and the various ways in which healthy eating messages are communicated in this context. This study acknowledges the growing popularity of healthy eating blogs as a means of accessing healthy eating information, and the growing use of healthy eating blogs by dietitians, as a means to communicate this information [12]. This growth highlights the need to consider the development of healthy eating blog guidelines to support dietitians in communicating appropriately and effectively in this context through the presentation of nutritional information online.

Communicating the purpose of each blog post was identified as a main characteristic of successful healthy eating blogs. Authors explicitly stated the purpose of the post in either the title, within the first few sentences of the first paragraph or through the use of heading. Hoffmann and Worrall (2004) highlighted the importance of conveying the intended purpose of educational materials to allow the reader to assess whether the material is of value [24]. Clearly conveying the purpose of the post through headings is strongly supported by advertising research that suggest that headings which offer a desired benefit and arouse curiosity are more likely to be engaging [43]. Additionally, headings that incorporate a desirable and believable action, like those identified in the healthy eating blogs included in this study, are also more likely to be engaging and read [43].

A distinguishable feature of all blogs, unlike websites, is the capacity for continuous conversation and communication, which is primarily facilitated by a comment box [21, 44]. Findings from this study highlighted varying participation levels of readers and authors communicating through the comment box. It has been reported that a comment box can provide insights into the views and perspectives of the reader, which can contribute to the development of posts that directly relates to the reader’s desire for information, ensuring their long-term readership and commitment [21, 43] [45]. However, despite the benefits of the comment box in facilitating communication, it is unclear what relationship there is between the author and the reader and what factors facilitate or impede that relationship.

This study also reported that a similar choice of writing style, vocabulary and layout were common characteristics within the structure of the blog posts. While there has been little empirical evidence investigating writing style and use of vocabulary in healthy eating blog posts, some studies have suggested that online health-related information aligns with eighth grade reading level and above, as compared to the recommended sixth-grade reading level [19, 20, 22, 25, 38, 39]. A review of patient education materials for rheumatic disease reported variability in writing style across resources, with readability of some resources aligned with an eighth grade reading level and above [19]. Similarly, a review of web-based colorectal cancer screening education also reported that the readability surpassed the recommended sixth-grade reading level [46]. This finding was also supported by Chen and Dunn (2015) who, after analysing 250 Australian-based websites containing online health information, reported that the average reading level also surpassed the recommended level [22]. While readability of blog posts was not analysed in this study, blog authors consistently used conversational language, short sentence structure and traditionally presented information using bullet-points.

The use of a consistent layout creates a sense of familiarity for the reader, allowing the reader to easily navigate through the information within each blog post [41]. Consistent with the literature, blogs included for analysis presented information in a predictable manner, with key messages and information appearing at the beginning of a post [24, 43]. Presenting key messages within the first paragraph is believed to effectively capture the reader’s attention, as the first paragraph is the most read part of any written material [43]. In addition to the location of key messages, the way they are communicated is also important. It has been suggested that positive key messages, compared to messages that are perceived to have a negative consequence or outcome, are more likely to capture the readers interest [47]. Interestingly, the blog posts included in this study focused on a positive aspect or outcome of food and nutrition in promoting health and wellbeing, rather than concentrating on a negative outcome.

Blog authors translated healthy eating knowledge in simple terms by generating practical recommendations and ideas for the reader to implement in their everyday life. These findings are supported by a study investigating health and fitness social media use in young adults [48]. The study identified that readers sought and valued practical information and identified the use of social media as a supportive means to encourage behaviour change through the communication of practical information, which then inspired and motivated healthier behaviours [48]. It has been reported that practical information communicated to readers by healthy eating blogs, including recipe ideas, tips to improve diet-related lifestyle issues and general healthy eating tips, are valued by readers and influence returned readership [7, 48]. The desire for practical information emphasises the importance of communicating procedural healthy eating information, rather than declarative [49]. While the latter describes the ability to recall and state facts, the former refers to the ability to apply facts in everyday life [49]. Previous research has emphasised the need for nutrition education to communicate procedural information, rather than only declarative education, which has traditionally been communicated [49–52]. However, while procedural knowledge seems to be advocated within the literature, further investigation is warranted to assess whether procedural knowledge facilitates behaviour change, especially in an online setting [53].

This study was subject to some limitations. First, despite conducting an extensive search in major search engines, healthy eating blogs were mainly identified through snowball sampling. There is no gold standard for identifying healthy eating blogs due to the changing nature of the blogosphere, hence it was assumed that if the blog did not appear in the first page of an engine search it is not frequently viewed [30–32]. Therefore, it is likely that not all healthy eating blogs that adhered to the inclusion criteria were captured by our search strategy. Seasonality is another consideration as this study was conducted through the festive season in Australia and therefore this may have influenced the content of blog posts. A similar investigation over another time period would identify whether seasonal factors influenced the identification of healthy eating blogs and post content. While the aim of the study was to investigate successful healthy eating blogs written by health professionals, the self-reported credentials were not verified, therefore questioning the assumption of evidenced-based information communicated within each blog. This study did not investigate the intended audiences of each healthy eating blog, a future direction for research in this area is to explore whether there are differences in how healthy eating messages are communicated for different sociodemographic groups. Additionally, it is recommended that future research also examines the reliability of nutrition information provided by healthy eating blogs.

### Recommendations

Blogs are fast becoming a popular medium to access and communicate healthy eating information [1, 6, 16, 38, 54]. Although more research is needed to assess the effectiveness and impact of healthy eating blogs on behavioural change, it is recommended that the dietetic profession embrace this medium as an avenue to disseminate current, evidence-based and practical healthy eating information. To do so, the development of blog guidelines may be useful in providing dietitians – and potentially health professionals in other disciplines - with a framework to create long-term successful blog posts. Guidelines should consider findings from this study which suggest that successful healthy eating blogs should clearly convey the purpose of each post, understand reader interest by clarifying their needs, maintain consistency of writing style, vocabulary and layout, and communicate nutrition messages in a variety of practical and simple ways.

## Conclusion

This study identifies the main features of a successful healthy eating blog, and the various ways in which healthy eating messages are communicated in this context. This study acknowledges the growing popularity of healthy eating blogs as a means of accessing healthy eating information, and the growing use of healthy eating blogs by dietitians, as a means to communicate this information. This growth highlights the need to consider the development of healthy eating blog guidelines to support dietitians in communicating appropriately and effectively in this context through the presentation of nutritional information online.

## Supplementary information

**Additional file 1: Table S1.** Comparison between healthy eating information from blog posts with recommendations from the Australian Dietary Guidelines (ADG).

## Data Availability

The datasets used and/or analysed during the current study are available from the corresponding author on reasonable request.

## Abbreviations

ADG: Australian Dietary Guidelines
APDs: Accredited Practicing Dietitians
HRWEF: The Health-Related Website Evaluation Form
SAM: Suitability Assessment of Material
