# Supplementary material for: Reframing healthy food choices: a content analysis of Australian healthy eating blogs
Source: BMC Public Health. 2019 Dec 19;19:1711. doi: 10.1186/s12889-019-8064-7 (PMC6923928; doi:10.1186/s12889-019-8064-7)
Supplement: Supplementary file 1 — Additional file 1: Table S1. Comparison between healthy eating information from blog posts with recommendations from the Australian Dietary Guidelines (ADG). [file 12889_2019_8064_MOESM1_ESM.docx]

**Appendix**

**Table 4. Comparison between healthy eating information from blog posts with recommendations from the Australian Dietary Guidelines (ADG).**

| **ID** | **Type of Post** | **ADG** | | | | | **Description of Post** | **Degree of consensus** | | | |
| --- | --- | --- | --- | --- | --- | --- | --- | --- | --- | --- | --- |
|  |  | **1^a^** | **2^a^** | **3^a^** | **4^a^** | **N^a^** |  | **2^b^** | **1^b^** | **0^b^** | **N^b^** |
| **E#1** | Recipe |  |  |  |  |  | Encourages variety, vegetable and legume consumption |  |  |  |  |
| **E#2** | Healthier Alternatives |  |  |  |  |  | Promote the consumption of higher quality carbohydrates. Promotes the use of a nutritional supplement. |  |  |  |  |
| **E#3** | Recipe |  |  |  |  |  | Ingredients are mainly discretionary. |  |  |  |  |
| **E#4** | Healthier Alternatives |  |  |  |  |  | Briefly encourages the inclusion of vegetables, but focuses on the use of a nutritional (protein) supplement for children. |  |  |  |  |
| **E#5** | Food swaps |  |  |  |  |  | Encouraged the consumption of saturated fats. |  |  |  |  |
| **E#6** | Recipe |  |  |  |  |  | Distortion of recommendations to suit opinion. |  |  |  |  |
| **E#7** | Tips |  |  |  |  |  | Nutrition related but not applicable. |  |  |  |  |
| **E#8** | Recipe |  |  |  |  |  | Encourages vegetables consumption. |  |  |  |  |
| **E#9** | Tips |  |  |  |  |  | Nutrition related but not applicable. |  |  |  |  |
| **E#10** | Recipe |  |  |  |  |  | Promotes variety and vegetable/legume consumption |  |  |  |  |
| **A#1** | Meal Ideas |  |  |  |  |  | Promotes wholefoods, primarily, vegetables. |  |  |  |  |
| **A#2** | Recipe |  |  |  |  |  | Promotes wholefoods. |  |  |  |  |
| **A#3** | Ingredient information |  |  |  |  |  | Nutrition related but not applicable. |  |  |  |  |
| **A#4** | Recipe |  |  |  |  |  | Promotes wholefoods. |  |  |  |  |
| **A#5** | Food Preparation Tips |  |  |  |  |  | Promotes wholegrain options and the addition of vegetables. |  |  |  |  |
| **A#6** | Recipe |  |  |  |  |  | Promotes wholefood and appropriate discretionary portion sizes. |  |  |  |  |
| **A#7** | Food Swaps |  |  |  |  |  | Promotes wholefood alternatives. |  |  |  |  |
| **A#8** | Recipe |  |  |  |  |  | Promotes wholefoods. |  |  |  |  |
| **A#9** | Recipe |  |  |  |  |  | Promotes wholefoods over discretionary foods. |  |  |  |  |
| **A#10** | Food Preparation Tips |  |  |  |  |  | Promotes wholefoods. |  |  |  |  |
| **A#11** | Recipe |  |  |  |  |  | Promotes the consumption of water over soft drink and caffeine. |  |  |  |  |
| **A#12** | Recipe |  |  |  |  |  | Promotes the consumption of vegetables. |  |  |  |  |
| **A#13** | Recipe |  |  |  |  |  | Promotes fruit consumption. |  |  |  |  |
| **A#14** | Recipe |  |  |  |  |  | Mainly promotes wholefoods. Promotes use of coconut oil as an alternative to olive oil. |  |  |  |  |
| **A#15** | Recipe |  |  |  |  |  | Primarily contains discretionary ingredients. |  |  |  |  |
| **A#16** | Opinion Piece |  |  |  |  |  | Promotes foods from all food groups. |  |  |  |  |
| **A#17** | Recipe |  |  |  |  |  | Promotes wholefoods. |  |  |  |  |
| **A#18** | Food Swaps |  |  |  |  |  | Promotes wholefoods and moderation. |  |  |  |  |
| **A#19** | Recipe |  |  |  |  |  | Promotes the consumption of vegetables. |  |  |  |  |
| **A#20** | Food Ideas |  |  |  |  |  | Promotes wholefoods over nutritional supplements. |  |  |  |  |
| **A#21** | Recipe |  |  |  |  |  | Promotes wholefoods and moderation. |  |  |  |  |
| **D#1** | Tips |  |  |  |  |  | Nutrition related but not applicable. |  |  |  |  |
| **D#2** | Food Tips |  |  |  |  |  | Promotes moderation and vegetable consumption. Implies 'good' and 'bad' vegetables. |  |  |  |  |
| **D#3** | Recipe |  |  |  |  |  | Promotes wholefoods. |  |  |  |  |
| **D#4** | Guest Interview |  |  |  |  |  | Nutrition related but not applicable. |  |  |  |  |
| **D#5** | Tips |  |  |  |  |  | Nutrition related but not applicable. |  |  |  |  |
| **D#6** | Recipe |  |  |  |  |  | Promotes whole foods. |  |  |  |  |
| **D#7** | Opinion Piece |  |  |  |  |  | Nutrition related but not applicable. |  |  |  |  |
| **D#8** | Guest Interview |  |  |  |  |  | Nutrition related but not applicable. |  |  |  |  |
| **D#9** | Recipe |  |  |  |  |  | Primarily contains discretionary ingredients. |  |  |  |  |
| **D#10** | Tips |  |  |  |  |  | Nutrition related but not applicable. |  |  |  |  |
| **D#11** | Food Tips |  |  |  |  |  | Promotes moderation. Distortion of recommendations to suit opinion. |  |  |  |  |
| **D#12** | Recipe |  |  |  |  |  | Promotes wholefoods. |  |  |  |  |
| **D#13** | Information |  |  |  |  |  | Nutrition related but not applicable. |  |  |  |  |
| **D#14** | Food Ideas |  |  |  |  |  | Promotes wholefoods. |  |  |  |  |
| **D#15** | Recipe |  |  |  |  |  | Promotes wholefoods. |  |  |  |  |
| **D#16** | Tips |  |  |  |  |  | Nutrition related but not applicable. |  |  |  |  |
| **D#17** | Tips |  |  |  |  |  | Nutrition related but not applicable. |  |  |  |  |
| **D#18** | Recipe |  |  |  |  |  | Primarily contains discretionary ingredients. |  |  |  |  |
| **D#19** | Tips |  |  |  |  |  | Nutrition related but not applicable. |  |  |  |  |
| **D#20** | Guest Interview |  |  |  |  |  | Nutrition related but not applicable. |  |  |  |  |
| **C#1** | Tips |  |  |  |  |  | Nutrition related but not applicable. |  |  |  |  |
| **C#2** | Recipe |  |  |  |  |  | Promotes moderation. Distortion of recommendations to suit opinion. |  |  |  |  |
| **C#3** | Benefits of nutrition and PA |  |  |  |  |  | Nutrition related but not applicable. |  |  |  |  |
| **C#4** | Recipe |  |  |  |  |  | Promotes wholefoods. |  |  |  |  |
| **C#5** | Meal Ideas |  |  |  |  |  | Promotes the consumption of vegetables. |  |  |  |  |
| **C#6** | Tips |  |  |  |  |  | Nutrition related but not applicable. |  |  |  |  |
| **C#7** | Recipe |  |  |  |  |  | Promotes wholefoods. |  |  |  |  |
| **C#8** | Recommendations |  |  |  |  |  | Nutrition related but not applicable. |  |  |  |  |
| **C#9** | Tips |  |  |  |  |  | Nutrition related but not applicable. |  |  |  |  |
| **C#10** | Tips |  |  |  |  |  | Nutrition related but not applicable. |  |  |  |  |
| **C#11** | Ingredient Information |  |  |  |  |  | Nutrition related but not applicable. |  |  |  |  |
| **C#12** | Tips |  |  |  |  |  | Nutrition related but not applicable. |  |  |  |  |
| **C#13** | Food Information |  |  |  |  |  | Promotes moderation. Distortion of recommendations to suit opinion. |  |  |  |  |
| **C#14** | Tips |  |  |  |  |  | Nutrition related but not applicable. |  |  |  |  |
| **B#1** | Recipe |  |  |  |  |  | Promotes moderation. |  |  |  |  |
| **B#2** | Meal Ideas |  |  |  |  |  | Promotes wholefoods. |  |  |  |  |
| **B#3** | Meal Ideas |  |  |  |  |  | Promotes wholefoods. |  |  |  |  |
| **B#4** | Recipe |  |  |  |  |  | Promotes wholefoods. |  |  |  |  |
| **B#5** | Recipe |  |  |  |  |  | Promotes wholefoods. |  |  |  |  |
| **B#6** | Recipe |  |  |  |  |  | Promotes wholefoods. |  |  |  |  |
| **B#7** | Recipe |  |  |  |  |  | Promotes vegetables consumption. |  |  |  |  |
| **B#8** | Recipe |  |  |  |  |  | Promotes vegetable and wholegrain alternatives. |  |  |  |  |
| **B#9** | Recipe |  |  |  |  |  | Promotes wholegrain alternatives. |  |  |  |  |
| **B#10** | Recipe |  |  |  |  |  | Promotes moderation. |  |  |  |  |
| **B#11** | Recipe |  |  |  |  |  | Promotes moderation. |  |  |  |  |

^a^ Refers to the Australian Dietary Guideline number

^b^ Refers to the level of consensus (*2 = information clearly aligns with recommendations. 1= information somewhat aligns. 0 = information does not align with recommendations)*
